# In vitro Selection and in vivo Testing of Riboswitch-inspired Aptamers

**DOI:** 10.21769/BioProtoc.4775

**Published:** 2023-07-05

**Authors:** Michael G. Mohsen, Ronald R. Breaker

**Affiliations:** 1 Department of Molecular, Cellular and Developmental Biology, Yale University, New Haven, CT 06511, USA; 2 Howard Hughes Medical Institute, Yale University, New Haven, CT 06511, USA; 3 Department of Molecular Biophysics and Biochemistry, Yale University, New Haven, CT 06511, USA

**Keywords:** Aptamer, Directed evolution, In vitro selection, Riboswitch, Synthetic biology

## Abstract

Engineered aptamers for new compounds are typically produced by using in vitro selection methods. However, aptamers that are developed in vitro might not function as expected when introduced into complex cellular environments. One approach that addresses this concern is the design of initial RNA pools for selection that contain structural scaffolds from naturally occurring riboswitch aptamers. Here, we provide guidance on design and experimental principles for developing riboswitch-inspired aptamers for new ligands. The in vitro selection protocol (based on Capture-SELEX) is generalizable to diverse RNA scaffold types and amenable to multiplexing of ligand candidates. We discuss strategies to avoid propagation of selfish sequences that can easily dominate the selection. We also detail the identification of aptamer candidates using next-generation sequencing and bioinformatics, and subsequent biochemical validation of aptamer candidates. Finally, we describe functional testing of aptamer candidates in bacterial cell culture.

Key features

Develop riboswitch-inspired aptamers for new ligands using in vitro selection.

Ligand candidates can be multiplexed to conserve time and resources.

Test aptamer candidates in bacterial cells by grafting the aptamer back onto its expression platform.

**Graphical overview**


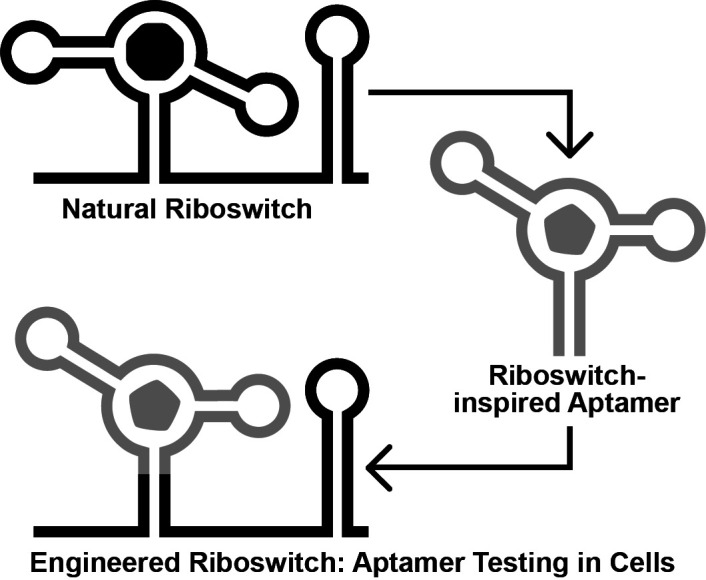


## Background

Aptamers are ligand-binding oligonucleotides that are becoming increasingly useful for broad applications in diagnostics, therapeutics, and synthetic biology (Keefe et al., 2010; Topp and Gallivan, 2010; Wang et al., 2019). Aptamers occur naturally in the context of riboswitches, where they monitor the concentration of a target ligand and manipulate the folding of an adjoining expression platform to control the expression of their associated genes (Sherwood and Henkin, 2016; Kavita and Breaker, 2023). Engineered aptamers that bind different ligands can be developed using a technique called in vitro selection (Ellington and Szostak, 1990; Tuerk and Gold, 1990). This process involves generating a large combinatorial pool of oligonucleotides, selecting for those that bind a target ligand, and amplifying the selected oligonucleotides. This process is repeated iteratively until aptamers for the target ligand are identified.

**Figure 1.**
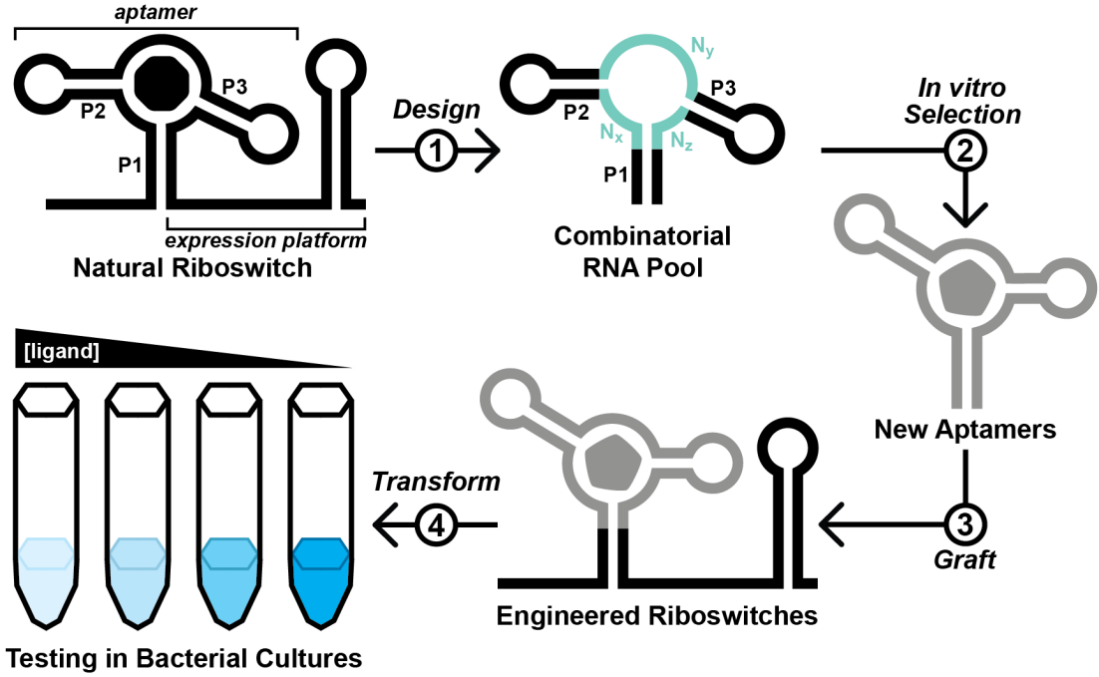
Overview of the Graftamer approach. First, a combinatorial RNA pool is designed by inserting regions of random RNA sequence (N_x_, N_y_, and N_z_) in between structural features of a natural riboswitch aptamer. The depicted riboswitch contains an aptamer with paired elements P1, P2, and P3. Second, in vitro selection is performed to develop riboswitch-inspired aptamers that bind ligands different than that of the natural riboswitch. Third, validated aptamers that maintain the structural features of the natural riboswitch are grafted back onto the expression platform of the natural riboswitch to construct engineered riboswitches. Fourth, plasmids containing engineered riboswitches positioned upstream of a reporter gene are transformed into a suitable model organism. In the depicted example, the riboswitch functions as an OFF switch. Increased ligand concentration reduces the expression of a *lacZ* reporter gene, resulting in a corresponding decrease in blue color in the presence of X-gal indicator.

Ideally, engineered aptamers could be useful for intracellular applications. However, the physiochemical conditions inside of a cell differ substantially from that of a test tube. Thus, aptamers developed in vitro might fail to perform inside of a cell, likely due to intrinsic factors such as the failure to reliably fold into the structure required to form the ligand binding pocket (Filonov et al., 2014). To address this, researchers have exploited the architectures of natural riboswitch aptamers to provide scaffolds for combinatorial RNA pools (Porter et al., 2017; Dey et al., 2022; Mohsen et al., 2023). We recently reported the Graftamer approach (Mohsen et al., 2023), in which engineered aptamers that contain a natural riboswitch scaffold are grafted back onto the natural expression platform (Figure 1). Using this approach, we developed aptamers for quinine and caffeine that retain the Guanine-I riboswitch (Mandal et al., 2003) scaffold from the initial combinatorial pool. These aptamers were each grafted back onto the expression platform of a *Bacillus subtilis xpt-pbuX* Guanine-I riboswitch. The resulting engineered quinine and caffeine riboswitches each display ligand-mediated gene regulation in *B. subtilis* cultures, indicating that the quinine and caffeine aptamers are functional in cells.

**Figure 2.**
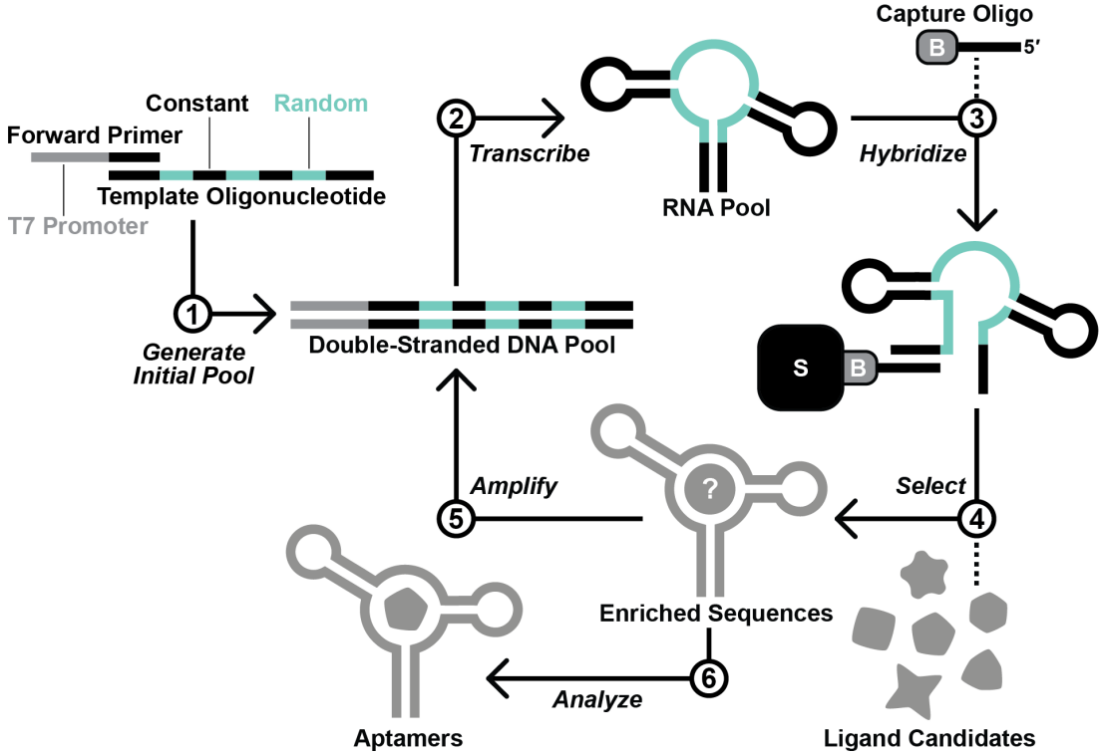
Overview of the in vitro selection scheme. First, the template oligodeoxynucleotide and forward primer are used to generate the initial DNA pool by primer extension. Second, in vitro transcription with T7 RNA polymerase is performed to produce the initial RNA pool. Third, the RNA pool is hybridized with a 3′-biotinylated DNA capture oligonucleotide. Fourth, RNA molecules that display affinity for a ligand candidate are isolated via a selection step. Fifth, the RNA molecules that survive the selection step are amplified by reverse transcription-polymerase chain reaction (RT-PCR). Steps 2–5 are repeated iteratively until the RNA pool is sufficiently enriched with functional aptamers. Finally, aptamer sequences are identified by sequencing and testing.

Here, we provide a protocol for this approach, beginning from the design and generation of the initial RNA pool. The selection process described herein is based on Capture-SELEX (Nutiu and Li, 2005; Stoltenburg et al., 2012; Yang et al., 2016; Lauridsen et al., 2018; Boussebayle et al., 2019), wherein the RNA pool is hybridized to a 3′-biotinylated DNA capture oligonucleotide, which itself is immobilized on a streptavidin-agarose column (Figure 2). In principle, RNA molecules that undergo a structural change upon binding a ligand are able to release from the capture oligonucleotide (Nutiu and Li, 2005). However, selfish molecules that slowly release from the capture oligonucleotide in a ligand-independent manner can jeopardize the selection (Mohsen et al., 2023). To counter the proliferation of selfish molecules, we recommend stringent washing and relatively short incubation times. Contamination between parallel lines of in vitro selection poses a threat as well. Thus, we suggest designing a different set of primers for each selection line performed in the same laboratory space. We anticipate that this protocol might have reduced utility for researchers pursuing aptamers of compounds that occur naturally in the target organism or that are unable to accumulate to appreciable intracellular concentrations (e.g., due to rapid efflux or metabolism, or an inability to permeate the cell wall or membrane). Nevertheless, we expect that this protocol will be broadly useful for improving best practices for in vitro selection and for increasing the likelihood of success for other researchers working to develop novel aptamers.

## Materials and reagents

**Biological materials**

1. *Bacillus subtilis* strain 1A1 (American Type Culture Collection)
2. *Escherichia coli* strain BW25113 (Coli Genetic Stock Center at Yale University)

**Reagents**

1. Agarose (IBI Scientific, catalog number: IB70071)
2. Boric acid (Sigma, catalog number: B0394)
3. Bromophenol blue (Sigma, catalog number: B5525)
4. Dimethylformamide (Sigma, catalog number: 319937)
5. Dithiothreitol (DTT) (American Bioanalytical, catalog number: AB00490)
6. Ethanol, 200 proof HPLC grade (Sigma, catalog number: 459828)
7. Ethylenediaminetetraacetic acid (EDTA) (Sigma, catalog number: E5134)
8. Glacial acetic acid (J.T. Baker, catalog number: 9508-33)
9. HEPES [(4-(2-hydroxyethyl)-1-piperazineethanesulfonic acid] (American Bioanalytical, catalog number: AB00892)
10. Magnesium chloride hexahydrate (MgCl_2_·6H_2_O) (J.T. Baker, catalog number: 2444-01)
11. Potassium chloride (KCl) (Sigma, catalog number: 746436)
12. Purple 100 base pair DNA ladder (New England Biolabs, catalog number: N0551S)
13. Purple gel loading dye (6×) (New England Biolabs, catalog number: B7024S)
14. Sodium acetate (NaOAc) (Sigma, catalog number: 241245)
15. Sodium hydroxide (NaOH) pellets (J.T. Baker, catalog number: 3722-01)
16. Spermidine (Sigma, catalog number: S2501)
17. Sucrose (Sigma, catalog number: S0389)
18. SuperScript III Reverse Transcriptase (Invitrogen, catalog number: 18080093)
19. T7 RNA Polymerase (purified in-house; equivalent to New England Biolabs, catalog number: M0251L)
20. Taq DNA polymerase (New England Biolabs, catalog number: M0273L)
21. Trizma (Tris) base (Sigma, catalog number: T6066)
22. TURBO DNase (2 U/μL) (Invitrogen, catalog number: AM2238)
23. Urea (Sigma, catalog number: U5378)
24. UreaGel 19:1 concentrate (National Diagnostics, catalog number: EC-830)
25. UreaGel buffer (National Diagnostics, catalog number: EC-835)
26. UreaGel system diluent (National Diagnostics, catalog number: EC-840)
27. X-gal (5-bromo-4-chloro-3-indolyl-β-D-galactopyranoside) (Cayman, catalog number: 16495)
28. Xylene cyanol FF (Sigma, catalog number: X-4126)

**Solutions**

1. 2 M KCl (see Recipes)
2. 5 M NaCl (see Recipes)
3. 3 M NaOAc (see Recipes)
4. 1 M MgCl_2_ (see Recipes)
5. 1 M spermidine (see Recipes)
6. 1 M Tris (pH 7.5 at ~20 °C) (see Recipes)
7. 1 M dithiothreitol (DTT) (see Recipes)
8. X-gal (100 μg/mL) in dimethylformamide (see Recipes)
9. 0.5 M EDTA (pH 8.0 at ~20 °C) (see Recipes)
10. Tris-borate-EDTA (TBE) (10×) (see Recipes)
11. Tris-acetate-EDTA (TAE) (50×) (see Recipes)
12. Transcription buffer (10×) (see Recipes)
13. Selection buffer (10×) (see Recipes)
14. Loading buffer (2×) (see Recipes)
15. Crush-soak buffer (see Recipes)

## Recipes

1. **2 M KCl**
  Add 14.9 g of KCl to a 100 mL flask. Add deionized H_2_O to 100 mL. Stir to mix. Filter-sterilize.
2. **5 M NaCl**
  Add 29.2 g of NaCl to a 100 mL flask. Add deionized H_2_O to 100 mL. Stir to mix. Filter-sterilize.
3. **3 M NaOAc**
  Add 24.6 g of NaOAc to a 100 mL flask. Add deionized H_2_O to 100 mL. Stir to mix. Filter-sterilize.
4. **1 M MgCl_2_**
  Add 9.5 g of MgCl_2_ to a 100 mL flask. Add deionized H_2_O to 100 mL. Stir to mix. Filter-sterilize.
5. **1 M spermidine**
  Add 145 mg of spermidine to a 1.5 mL microcentrifuge tube. Add 1 mL of dH_2_O. Vortex to mix.
  Add 1 mL of deionized, sterile H_2_O (dH_2_O).
6. **1 M Tris (pH 7.5 at ~20 °C)**
  Add 121.1 g of Tris to a 1 L flask. Add deionized H_2_O to ~900 mL. While stirring, adjust pH with hydrochloric acid to pH 7.5. Add deionized H_2_O to 1 L. Autoclave.
7. **1 M dithiothreitol (DTT)**
  Add 154 mg of DTT to a 1.5 mL microcentrifuge tube. Add 1 mL of dH_2_O. Vortex to mix.
8. **X-gal (100 μg/mL) in dimethylformamide**
  Add 100 mg of X-gal to a 1.5 mL microcentrifuge tube. Add 1 mL of dimethylformamide. Vortex to mix.
9. **0.5 M EDTA (pH 8.0 at ~20 °C)**
  Add 186.1 g of EDTA to a 1 L flask. Add deionized H_2_O to ~800 mL. Stir to mix. Add 15 g of NaOH pellets. Stir to mix. While stirring, adjust pH with 10 N sodium hydroxide to pH 8.0. Add deionized H_2_O to 1 L. Autoclave.
10. **TBE (10× concentrated, 4 L)**
  To a 4 L flask, add 432 g of Tris base, 220 g of boric acid, 14.9 g of EDTA, and deionized H_2_O to 4 L. Stir to mix. Filter particulates. Sterilize by autoclaving. The final solution is a 10× concentration buffer containing 0.9 M Tris, 0.9 M borate, and 10 mM EDTA pH 8.0 at ~20 °C.
11. **TAE (50× concentrated, 1 L)**
  To a 1 L flask, add 242 g of Tris base, deionized H_2_O to ~800 mL, 57.1 mL of glacial acetic acid, and 100 mL of 0.5 M EDTA pH 8.0 at ~20 °C. Add deionized H_2_O to 1 L. Filter particulates. Sterilize by autoclaving. The final solution is a 50× concentrated buffer containing 2 M Tris, 1 M acetate, and 50 mM EDTA pH 8.0 at ~20 °C.
12. **Transcription buffer (10× concentrated, 1 mL)**
  Mix 150 μL of 1 M MgCl_2_, 20 μL of 1 M spermidine, 500 μL of 1 M Tris (pH 7.5 at ~20 °C), 50 μL of 1 M DTT, and 280 μL of dH_2_O in a 1.5 mL microcentrifuge tube. 1× mixture: 150 mM MgCl_2_, 20 mM spermidine, 500 mM Tris (pH 7.5 at ~20 °C), and 50 mM DTT. Store at -20 °C
13. **Selection buffer (10× concentrated, 100 mL)**
  Mix 4.77 g of HEPES, 50 mL of 2 M KCl, 1 mL of 1 M MgCl_2_, and 35 mL of deionized H_2_O together in a flask containing a magnetic stir bar. Adjust the pH of the solution to 7.4 by adding pre-weighed NaOH pellets while stirring. Once a pH of 7.4 is attained, remove any remaining NaOH pellets with a clean spatula. Dry and weigh the pellets. After calculating the amount of NaOH required to adjust the pH (typically ~70 mM), add a volume (typically ~0.6 mL) of 5 M NaCl to the solution to bring the total [Na^+^] to 100 mM. Add deionized H_2_O to adjust the final volume of the solution to 100 mL. Filter-sterilize the resulting solution. The final solution is a 10× concentrated selection buffer containing 200 mM HEPES, 1 M KCl, 30 mM NaCl (total [Na^+^]: 100 mM), and 10 mM MgCl_2_, pH 7.4 at ~20 °C [1×: 20 mM HEPES, 100 mM KCl, 3 mM NaCl (total [Na^+^]: 10 mM), and 1 mM MgCl_2_]. HEPES is light-sensitive, so store this buffer in a darkened compartment (cabinet, refrigerator, etc.).
14. **Loading buffer (2× concentrated, 40 mL)**
  Add 44 g of urea to a flask and add 28 mL of dH_2_O. Stir with gentle heat until urea is dissolved. Add 8 g of sucrose, 20 mg of bromophenol blue, 20 mg of xylene cyanole FF, 0.4 mL of 10% SDS, and 4 mL of 10× TBE. Stir with gentle heat until sucrose is dissolved. Store at 4 °C.
15. **Crush-soak buffer**
  To a 1 L flask, add 40 mL of 5 M NaCl, 10 mL of 1 M Tris (pH 7.5 at ~20 °C), and 2 mL of 0.5 M EDTA (pH 8.0 at ~20 °C). Add deionized H_2_O to 1 L. Autoclave.

**Laboratory supplies**

1. 0.5 mL low adhesion microcentrifuge tubes (USA Scientific, catalog number: 1405-2600)
2. 1.5 mL low adhesion microcentrifuge tubes (USA Scientific, catalog number: 1415-2600)
3. 8-strip 0.2 mL PCR tubes (Dot Scientific, catalog number: 401)
4. Gel-loading pipette tips (VWR, catalog number: 76321-828)
5. Individual PCR tubes 8-tube strip, clear (Bio-Rad, catalog number: TLS0801)
6. iTaq Universal SYBR Green Supermix (Bio-Rad, catalog number: 1725121)
7. Luer-Lok 3 mL syringe (BD, catalog number: 309657)
8. Micro Bio-Spin^TM^ chromatography columns (Bio-Rad, catalog number: 7326204)
9. Optical flat 8-cap strips for 0.2 mL tube strips (Bio-Rad, catalog number: TC20803)
10. Pierce^TM^ Streptavidin agarose (Thermo Scientific, catalog number: 20353)
11. PrecisionGlide needle 27 G × 1/2 (BD, catalog number: 305109)
12. QIAquick PCR Purification kit (Qiagen, catalog number: 28104)
13. Sterile pipette tips with filters (filters help to avoid contamination)
14. Vivaspin 500 molecular weight cutoff 10 kDa (Cytiva, catalog number: 28932225)

## Equipment

1. Analytical balance (Mettler Toledo, catalog number: AG285)
2. Cary 60 UV-Vis spectrophotometer (originally Varian, now Agilent)
3. CFX Opus 96 Real-Time PCR System (Bio-Rad, catalog number: 12011319)
4. Geiger counter (Ludlum)
5. Gel Doc Go Gel imaging system (Bio-Rad, catalog number: 12009077)
6. Handheld shortwave (254 nm) ultraviolet lamp (UVP, catalog number: UVG-65)
7. Innova 42R shaking incubator (Eppendorf, catalog number: M1335-0004)
8. Mastercycler Nexus GX2 (Eppendorf, catalog number: 6336000015)
9. Milli-Q Advantage A10 (Millipore-Sigma, catalog number: Z00Q0V0WW)
10. Mini centrifuge (Thermo Fisher, catalog number: 75004061)
11. NanoDrop 8000 spectrophotometer (Thermo Fisher, catalog number: ND-8000)
12. Orion Star A211 Benchtop pH meter (Thermo Fisher, catalog number: STARA2110)
13. Phosphorimager Typhoon FLA 9500 (originally GE Healthcare, now Cytiva)
14. PowerPac HV power supply (Bio-Rad, catalog number: 1645056)
15. Slab Gel Dryer Model 583 (Bio-Rad, catalog number: 165-1745)
16. Sorvall Legend Micro 21R refrigerated centrifuge (Fisher Scientific, catalog number: 75002446)
17. Speed Vac (originally Thermo-Savant, now Thermo Fisher)
18. Standard set of pipettes that can transfer volumes in the range of 1–1,000 μL
19. Synergy Neo2 Multimode Reader (originally BioTek, catalog number: 1351000, now Agilent)
20. Vortex-Genie 2 (Scientific Industries, catalog number: SI-0236)

## Software

1. R2R version 1.0.6, 2018-12-10 (https://sourceforge.net/projects/weinberg-r2r/)
2. CMfinder version 0.4.1.18, 2019-04-22 (https://sourceforge.net/projects/weinberg-cmfinder/)
3. Scripts *toTally.py* and *selfishCluster.py*, 2023-01-09 (Mohsen et al., 2023)

## Procedure

- **Pool design and generation**
  1. Choose a riboswitch to exploit as a basis for initial RNA pool design. In the absence of other options, the Guanine-I riboswitch class (Mandal et al., 2003) contains a malleable scaffold that can be exploited for this protocol. Key considerations:
    - The riboswitch aptamer has conserved tertiary contacts.
    - A single stem (called P1, Figure 1) encloses the entire aptamer domain.
    - Crystal structure data are available.
    - The riboswitch is present in a model organism (e.g., *E. coli* or *B. subtilis*) that the researcher can culture with relative ease.
  2. Determine which nucleotides to randomize.
    - Based on crystallographic data, determine which nucleotides interact with the natural ligand.
    - Randomize nucleotides that interact with the natural ligand, as well as nucleotides that are not required to form conserved tertiary structural interactions. A greater number of randomized nucleotides provides a larger sequence space, though we have performed successful selections with as few as 23 randomized nucleotides.
  3. Design a 3′-biotinylated DNA capture oligonucleotide.
    - The capture oligo should be 12–18 nucleotides in length and should contain 10–15 nucleotides of complementarity with a constant region in the RNA pool.
    - The capture oligo can be designed to compete with the P1 stem of the RNA pool.
    - The capture oligo should contain a 3′-biotin or 3′-biotin-triethylene glycol (TEG) modification. The TEG spacer provides additional space between the RNA/capture oligo hybrid and the streptavidin/biotin complex. We have performed successful selections using capture oligos with and without the TEG spacer.
  4. Design primer-binding regions and primers for polymerase chain reaction (PCR).
    - Design forward and reverse primer-binding regions into the RNA pool that have roughly the same melting temperature (T_m_). Avoid natural sequences from common laboratory model organisms because this can lead to amplification of nucleic acid contaminants.
    - Design a forward primer (sense sequence) that starts with a T7 RNA polymerase (RNAP) promoter sequence at the 5′ end. The T7 RNAP promoter sequence with two additional G nucleotides at the 3′ terminus (for increased transcription efficiency) is as follows: TAATACGACTCACTATAGG. RNA transcribed from this template will start with two G nucleotides. Add the sense DNA sequence corresponding to the forward primer-binding region from the RNA pool after these two G nucleotides.
    - Design a primer (*reverse primer*) that is the reverse complement DNA sequence of the 3′ primer-binding region from the RNA pool.
  5. Design an oligodeoxynucleotide template pool.
    - Design a single-stranded reverse complement DNA that contains ~15 base pairs of overlap with the forward primer (not including the T7 RNAP promoter sequence) (Figure 2, *Generate Pool Generation*). This strand contains randomized nucleotides (N). Hand-mixed phosphoramidites provide an even distribution between all four nucleotides, but machine mixing is typically more cost effective and is sufficient for this selection protocol.
  6. Order and purify custom oligodeoxynucleotides.
    - Longer strands such as the template pool should be ordered at a synthesis scale (≥ 200 nmol) that ensures a sufficient quantity of full-length material. Shorter strands such as forward primer, reverse primer, and capture oligonucleotide can be ordered at the smallest synthesis scale.
    - We typically order custom oligodeoxynucleotides with standard desalting and purify in house by denaturing (8 M urea) 10% polyacrylamide gel electrophoresis (PAGE). Alternatively, oligodeoxynucleotides could be ordered with PAGE purification.
  7. Synthesize the generation zero (G0) double-stranded DNA template by primer extension.
    - To a 0.2 mL tube, add 100 pmol of reverse complement template DNA, 150 pmol of forward primer, and dH_2_O to 30 μL.
    - Incubate at 90 °C for 1 min. Cool at room temperature for 3 min.
    - Add 5 μL of 10 mM dNTPs, 10 μL of 5× first strand buffer (provided by manufacturer), 2.5 μL of 0.1 M DTT, and 2.5 μL of SuperScript III reverse transcriptase (RT).
    - Incubate at 55 °C for 1 h.
    - Heat-inactivate RT by incubating at 75 °C for 15 min.
  8. Synthesize the generation zero (G0) RNA pool via in vitro transcription.
    - Set up the following in vitro transcription reaction in a 0.5 mL tube:
      **Table:** 

Component	Quantity
10× transcription buffer	10 μL
10 mM NTPs	20 μL
dsDNA G0 template	12.5 μL (~25 pmol)
dH_2_O	55 μL
T7 RNA polymerase	2.5 μL
      Optional: Scale up in vitro transcription by setting up multiple reactions to sample a larger sequence space.
    - Incubate at 37 °C for 2 h or until a white precipitate (Mg_2_P_2_O_7_) is visible at the bottom of the tube (sometimes overnight incubation is required).
    - During this long incubation, prepare a denaturing (8 M urea) 10% polyacrylamide gel following the protocol provided with National Diagnostics UreaGel reagents. If possible, use a comb with wells that can each accommodate a ~200 μL sample but that are not so large that the band would be diffuse.
    - Add 1 μL of TURBO DNase. Incubate at 37 °C for 10 min.
    - Add 101 μL of 2× loading buffer. Load the entire sample in one well. If a ~200 μL sample cannot be accommodated by a single well, the RNA can alternatively be concentrated using an RNA clean-up kit or by precipitation with ethanol. In this case, add a corresponding quantity of 2× loading buffer to the concentrated RNA solution.
    - Run the gel until the xylene cyanol marker has migrated ~5–10 cm from the well, to adequately separate the RNA band from DNA template and from truncated in vitro transcription products. Remove the gel from the glass plates and cover both sides with plastic wrap.
    - Visualize the RNA band by ultraviolet (UV) shadowing. In a dark room, place the gel on top of an uncut thin layer chromatography (TLC) silica gel sheet (with fluorescence indicator 254 nm). Shine a handheld UV lamp (short wavelength) on the gel. The RNA band appears as a dark band distinct from the dye band. However, if insufficiently separated, the RNA band might overlap with the xylene cyanol band. Using a marker, draw a box on the plastic wrap to indicate the location of the band. Work quickly to minimize UV light exposure to the RNA. Caution: Avoid personal exposure to UV light.
    - Excise the marked band with a razor blade. Exclude the bottom and top 20% of the band to avoid propagating unwanted sequences with different lengths than the original pool design.
    - Transfer the excised gel piece to a pre-weighed 1.5 mL tube. Determine the weight of the excised gel piece. Crush the gel using a small pestle.
    - Add 2× v/w crush-soak buffer relative to the weight of the excised gel piece. For example, if the gel piece weighs 0.2 g, add 0.4 mL of crush-soak buffer. Vortex briefly and then incubate at room temperature with rotation for at least 30 min (can also be incubated at 4 °C with rotation).
    - Transfer the solution to a Spin-X filtration column. A benchtop centrifuge operated at maximum speed for ~1 min can be used to filter gel particles from the solution.
    - Approximate the buffer volume remaining after filtration using a pipette. Add 0.1 volumes of 3 M NaOAc and 2.5 volumes of cold (-20 °C) ethanol (100%). Optionally incubate at -20 °C for at least 20 min or overnight.
    - Centrifuge at 17,000× *g* for 15 min at 4 °C.
    - Aspirate the supernatant using a pipette, taking care not to disturb the pellet. If the quantity of RNA is sufficient, the pellet might be observed at the bottom of the tube, positioned in the direction of centrifugal force.
    - Dry the pellet by centrifugal evaporation (speed-vac) on medium heat for 5 min or until dry. Alternatively, the pellet can be air dried.
    - Resuspend the pellet in 50 μL of dH_2_O. Quantitate the concentration of the resulting solution using a NanoDrop spectrophotometer.
    **Figure 3.**
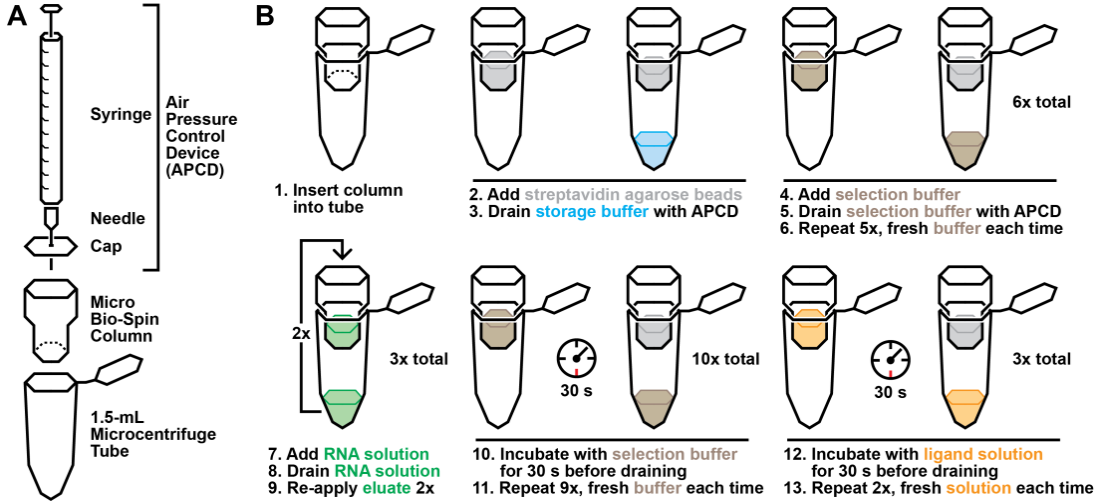
Graphical overview of the selection protocol. A. Laboratory materials required to perform the selection. B. Graphical summary of the selection procedure described in step B2.
- **In vitro selection**
  1. Hybridize RNA pool to capture oligonucleotide.
    - Prepare the following mixture in a 0.5 mL or 1.5 mL tube:
      **Table:** 

Component	Quantity
10× selection buffer	10 μL
RNA pool	*x* μL*
Capture oligonucleotide (10 μM)	*y* μL**
dH_2_O	90 - *x - y* μL
      *For round one, input 100–1,000 pmol RNA. For subsequent rounds, add 1–10 pmol RNA.
      **Add a 10× molar excess of capture oligonucleotide relative to RNA pool input.
    - Incubate the tube at 90 °C for 1 min and then allow to cool at room temperature for at least 5 min.
  2. Selection:
    - While the RNA-capture oligonucleotide solution is cooling, prepare a column for selection. Add 100 μL of streptavidin-agarose to a Micro Bio-Spin column using a P1000 pipette (the wider tip openings transfer the bead solution more accurately). Place the column in a 1.5 mL tube.
    - Prepare an air pressure control device (Figure 3A). Attach a 27 G × 1/2 needle to a 3 mL syringe. Then, poke the needle through the center of a Micro Bio-Spin cap. Pull the syringe plunger until it is fully extended.
    - Holding the column in one hand and the air pressure control device in the other, place the cap of the device on top of the column with sufficient pressure to create a seal, without shutting the tube. Apply pressure by pressing down on the syringe plunger. This should drain the storage buffer into the collection tube, while the streptavidin-agarose beads remain in the column.
    - Wash the column with 100 μL of 1× selection buffer six times to equilibrate the column in selection buffer (Figure 3B). Each wash is executed by gently pipetting 100 μL of buffer onto the column resin and subsequently using the air pressure control device to drain the buffer. Two consecutive washes can be collected in a single 1.5 mL tube, after which the column should be transferred to a new collection tube. After six washes, transfer the column to a new 1.5 mL collection tube.
    - Briefly centrifuge the RNA-capture oligonucleotide solution (after cooling for at least 5 min) and apply the entire 100 μL solution to the column. Use the air pressure control device to push the solution through the resin to the collection tube. To maximize the quantity of biotinylated capture oligonucleotide bound to the streptavidin column, re-apply the eluate to the column two additional times.
    - Wash the column with 100 μL of 1× selection buffer 10 times to remove RNA molecules retained by nonspecific interactions. In this case, each wash is performed by gently pipetting 100 μL of 1× selection buffer on top of the column resin and then using the air pressure control device to push the solution through, such that it saturates the resin but does not go through into the collection tube. After incubating for 30 s, use the air pressure control device to drain the solution into the collection tube.
    - Incubate three times with a solution of the chosen ligand candidate(s) in 1× selection buffer for 30 s. It is critical that the incubations with ligand solution are performed identically to the washes described in the preceding step, with the only difference being the presence of the ligand candidates.
    - Combine the ligand pool eluates and transfer to a Vivaspin 10 kDa molecular weight cutoff column. The mass of RNA pool molecules is expected to be > 10 kDa and should be retained by the column.
    - Centrifuge at 12,000× *g* for 15 min at room temperature (~20 °C).
    - Discard flowthrough. Add 300 μL of dH_2_O to the column and centrifuge again at 12,000× *g* for 15 min at room temperature.
    - Recover the concentrated RNA (typically < 15 μL) by aspirating with a gel-loading tip.
  3. Synthesize complementary DNA (cDNA) by reverse transcription.
    - During the first round of selection, prepare a generation zero (G0) marker. In parallel with the steps described below, perform reverse transcription using 2 pmol of the initial RNA pool.
    - To a 0.5 mL tube, add up to 12 μL of the concentrated RNA, 1 μL of 10 mM dNTPs, and 1 μL of 2 μM reverse primer (2 pmol). If the total volume of the concentrated RNA is < 12 μL, add dH_2_O to a total volume of 14 μL. Store any remaining RNA in excess of 12 μL at -20 °C for archiving.
    - Incubate the mixture at 65 °C for 5 min and then immediately incubate on ice for at least 1 min.
    - Briefly centrifuge and then add 4 μL of 5× first strand buffer (provided by manufacturer), 0.1 M DTT (provided by manufacturer), and 1 μL of SuperScript III RT.
    - Incubate at 55 °C for 60 min.
    - Incubate at 70 °C for 15 min to heat-inactivate RT.
  4. Quality control and scouting with quantitative polymerase chain reaction (qPCR)
    - Set up the following two reactions in 0.2 mL tubes:
      **Table:** 

Component	cDNA	Control
iTaq master mix	10 μL	10 μL
Forward + reverse primers (mixed, 4 μM each)	2 μL	2 μL
cDNA (from RT reaction)	2 μL	-
dH_2_O	6 μL	8 μL
    - Pipette-mix and briefly centrifuge the tubes ensuring that there are no bubbles, which can reflect light in unexpected ways and interfere with the analysis.
    - Set the qPCR program as follows:
      i. 95 °C, 1 min 45 s (initial denaturation)
      ii. 95 °C, 15 s (denaturation)
      iii. *x* °C, 30 s (annealing, *x* is annealing temperature)
      iv. 68 °C, 30 s (extension)
      v. Fluorescence measurement
      vi. Repeat steps ii–v 40×
*Note: Annealing temperature can be determined using the NEB Tm Calculator (accessible at tmcalculator.neb.com)*
    - Inspect the resulting fluorescence curves. The control sample often displays an increase in fluorescence intensity after 30–35 cycles, which might indicate amplification of a contaminant. If there is no difference between the cDNA and control curves, or if the curves begin to increase within only a few cycles of each other, the selection might be compromised by contamination.
    - Determine the number of PCR cycles to perform. This can be done manually by visually inspecting the fluorescence curves. The ideal number of cycles is just after the fluorescence curve for the cDNA sample has plateaued, indicating that amplification is near completion without over-amplification, which can result in undesired PCR products. Ideally, there should be no detectable fluorescence from the control sample at this chosen cycle number. Do not choose a cycle number greater than 30, as this is likely to lead to unwanted amplification of artifacts or contaminants.
  5. Amplify cDNA by PCR.
*Note: During the first selection round, continue the preparation of a G0 marker in parallel with the steps described below. Use 5 μL of the G0 reverse transcription reaction instead of the G1 reverse transcription reaction.*
    - Prepare a PCR reaction in a thin-walled 0.2 mL tube as illustrated below. One to three 50 μL PCR reactions can be prepared and performed simultaneously.
      **Table:** 

Component	Quantity
dH_2_O	34.5 μL
Standard Taq buffer	5 μL
10 mM dNTPs	1 μL
4 μM forward + reverse primers	5 μL
cDNA	5 μL
Taq DNA polymerase	0.5 μL
    - Using the chosen number of cycles, set up and run a PCR program as follows:
      i. 95 °C, 1 min 45 s (initial denaturation)
      ii. 95 °C, 15 s (denaturation)
      iii. *x* °C, 30 s (annealing, *x* is annealing temperature)
      iv. 68 °C, 30 s (extension)
      v. Repeat steps ii–v *y* times (*y* is the chosen number of cycles)
      vi. 68 °C, 2 min (final extension)
      vii. Hold at 10 °C
    - Take a 5 μL aliquot from the completed PCR reaction and mix it with 1 μL of 6× purple loading dye in a new tube.
    - To prepare the G0 marker, add 10 μL of 6× purple loading dye to the PCR tube and optionally transfer to a 1.5 mL tube for easier storage at 4 °C or -20 °C.
    - Prepare a 1.5% agarose gel by dissolving 0.6 g of agarose in 40 mL of TAE in a 250 mL Erlenmeyer flask. Microwave the solution in bursts with frequent stirring until the agarose is completely dissolved. Just before casting the gel, add 2 μL of ethidium bromide and mix by swirling.
    - Load 5 μL each of 100 base pair ladder, G0 marker, and the PCR solution from the current generation in consecutive lanes.
    - Run the gel at 115 V for 20 min. Visualize the resulting gel using a Bio-Rad Gel Doc Go Gel imaging system or an equivalent instrument. The bands resulting from the G0 marker and from the current generation should appear to have migrated the same distance, indicating that the amplicons originated from the pool and not from a contaminant.
    - Purify the PCR product using a QIAquick PCR purification kit or equivalent.
    - Quantitate the concentration of DNA using a NanoDrop spectrophotometer. Convert the concentration from ng/μL to μM by dividing by the approximate molecular weight of the DNA construct.
  6. Synthesize the RNA pool for the subsequent generation by in vitro transcription.
    - Set up the following in vitro transcription reaction in a 0.5 mL tube:
      **Table:** 

Component	Quantity
10× transcription buffer	10 μL
10 mM NTPs	20 μL
dsDNA from PCR	*x* μL (10 pmol)
dH_2_O	67.5 - *x* μL
T7 RNA polymerase	2.5 μL
    - Follow the protocol for in vitro transcription from section A.
  7. Iterative rounds of selection:
    - Perform iterative rounds of selection until the pool is sufficiently enriched. After the first few rounds, the researcher can optionally decide to decrease the concentration of ligand candidates to apply additional selection pressure. Another option that can be applied concurrently is to decrease the quantity of input RNA in later selection rounds.
    - Typically, the RNA pool will be sufficiently enriched after 8–12 rounds of selection. Enrichment can be assessed by an elution profile, which is performed similarly to the selection process described above.
- **Elution profile**
*Note: This step involves handling radioactive materials (^32^P). If the researcher’s laboratory is not equipped to handle radioactive materials, the entire process could be performed with unlabeled RNA. In this case, qRT-PCR could alternatively be used to determine relative quantities of RNA eluted with each ligand candidate.*
  1. Prepare the following mixture in a 0.5 or 1.5 mL tube:
    **Table:** 

Component	Quantity
10× selection buffer	10 μL
5′ ^32^P-labeled RNA pool	*x* μL (~50,000 counts per minute)
Capture oligonucleotide (10 μM)	1 μL
dH_2_O	89 - *x* μL
  2. Incubate the tube at 90 °C for 1 min and then allow to cool at room temperature for at least 5 min.
  3. While the RNA-capture oligonucleotide solution is cooling, prepare a column for the elution profile. Add 100 μL streptavidin-agarose to a Micro Bio-Spin column using a P1000 pipette. Place the column in a 1.5 mL tube.
  4. Prepare an air pressure control device: attach a needle to a 3 mL syringe. Then, poke the needle through the center of a Micro Bio-Spin cap. Pull the syringe plunger until it is fully extended.
  5. Holding the column in one hand and the air pressure control device in the other, place the cap of the device on top of the column with sufficient pressure to create a seal, without shutting the tube. Apply pressure by pressing down on the syringe plunger. This should drain the storage buffer into the collection tube, while the streptavidin-agarose beads remain in the column.
  6. Wash the column with 100 μL of 1× selection buffer six times to equilibrate the column in selection buffer. Each wash is executed by gently pipetting 100 μL of buffer onto the column resin and subsequently using the air pressure control device to drain the buffer. Two consecutive washes can be collected in a single 1.5 mL tube, after which the column should be transferred to a new collection tube. After six washes, transfer the column to a new 1.5 mL collection tube.
  7. Briefly centrifuge the RNA-capture oligonucleotide solution (after cooling for at least 5 min) and apply the entire 100 μL solution to the column. Use the air pressure control device to push the solution through the resin to the collection tube. To maximize the quantity of biotinylated capture oligonucleotide bound to the streptavidin column, re-apply the eluate to the column two additional times. After applying and eluting the solution three times, label the tube containing the final eluate as *Unbound RNA*.
  8. Incubate the column with 100 μL of 1× selection buffer for 30 s to remove RNA molecules retained by nonspecific interactions. Each incubation is performed by gently pipetting 100 μL of 1× selection buffer on top of the column resin and then using the air pressure control device to push the solution through, such that it saturates the resin but does not go through into the collection tube. After incubating for 30 s, use the air pressure control device to force the solution through into the collection chamber. Repeat this step until eluates reach background radiation, as assessed by a Geiger counter (typically 6–8 incubations). Collect each eluate in a separate tube.
  9. Incubate the column with a 100 μL solution of one of the chosen ligand candidates in 1× selection buffer. It is critical that the incubations are performed identically to the negative selection steps above, with the only difference being the presence of the ligand candidate. Repeat this step two times, collecting each eluate in a separate tube. If you performed selection with only one ligand candidate, the elution profile, skip to step C12.
  10. Incubate the column with 100 μL of 1× selection buffer for 30 s to remove the previous candidate ligand as well as RNA molecules retained by the column. Repeat this step two additional times before applying the solution with the next candidate.
  11. Incubate the column with a 100 μL solution of the next ligand candidate in 1× selection buffer three times. Repeat this step for all ligand candidates, washing three times with 1× selection buffer in between candidates.
  12. After all compounds have been assayed, use a pen to mark a grid on a piece of filter paper. The grid should be able to accommodate all samples. Pipette 1 μL of each sample in the center of each square marked by the gridlines. Allow the filter paper to air dry before covering it in plastic wrap.
  13. Expose the filter paper to a phosphor screen overnight and image the following day using a Typhoon phosphorimager. Note whether an increase in signal is observed in eluates with any of the compounds relative to the eluates without compound (see Figure 4).
  14. If selection was performed with multiple ligand candidates, repeat the elution profile, this time reversing the order of the ligand candidates. Signals usually fade over the course of the elution profile as less radiolabeled RNA remains on the column. This can sometimes provide a false positive signal for ligand candidates that are assayed earlier in the experiment.
  15. If the RNA pool appears to respond to one or more of the ligand candidates, continue on to Section D. Otherwise, more selection is required to further enrich the RNA pool.
    **Figure 4.**
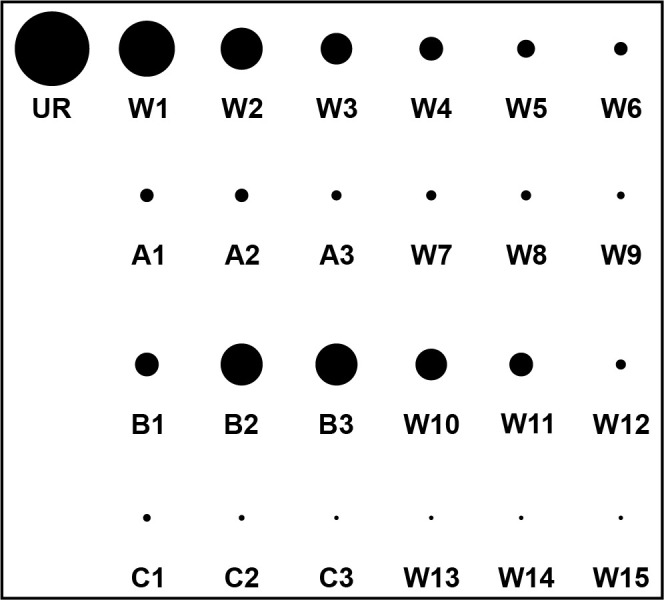
Illustration of a hypothetical elution profile readout that would be performed after a selection with three ligand candidates: A, B, and C. The eluate containing unbound RNA (UR) is followed by six washes with selection buffer (W1–W6), after which radiation levels are close to background radiation. Eluates containing compound A (A1–A3), compound B (B1–B3), and compound C (C1–C3) are each followed by three washes with selection buffer. Based on this result, the aptamer or RNA pool would appear to respond to compound B.
- **Identification of candidate sequences using next generation sequencing and bioinformatics**
  1. Preparation of DNA pools for next-generation sequencing is better covered in other protocols. Briefly, prepare an aliquot of the desired DNA library (at least 50 ng). We typically submit samples to a sequencing core (Yale Center for Genomic Analysis) where an Illumina NovaSeq is used to perform next generation sequencing with a read depth of ~40 million reads. Paired-end reads are sequenced with a read length of 150 base pairs. Ensure that the read length is greater than the length of the DNA pool including the T7 RNAP promoter sequence.
  2. After obtaining the sequencing data files (.fastq.gz format), use *toTally.py* to count the number of reads for each unique sequence and rank them according to abundance in tab-separated values (.tsv) format.
    $ ./toTally.py -i <path/to/file1.fastq.gz> -j <path/to/file2.fastq.gz>
    -5 <fwdPrimerSequence> -3 <revPrimerSequence>
  3. Generate putative classes for the top-ranked sequences using *selfishCluster.py*. This program outputs several fasta (.fa) files for each candidate. The 5′ and 3′ input sequences here are the primer binding regions of the RNA library (sense sequence). With default parameters, the output will be five fasta files. There will be fewer output files if two or more of the top five ranked sequences belong to the same class.
    $ ./selfishCluster.py -i <file.tsv> -5 <5' constant region> -3 <3' constant region>
  4. Use CMfinder to generate a list of conserved secondary structure motifs for each class. This step will generate many Stockholm (.sto) files. Many of these motifs represent a small portion of the full-length RNA.
    $ ./cmfinder-0.4.1.18/bin/cmfinder04.pl -skipClustalw -combine <file.fa>
  5. Use R2R to draw the conserved motifs outputted by CMfinder. If it is not possible to identify which .sto file contains the full-length RNA, it might be necessary to use R2R to draw all of them and inspect each output (.pdf) manually.
    $ ./R2R-1.0.6/src/r2r -GSC-weighted-consensus <fileName.sto> <fileName_cons.sto>
    3 0.97 0.9 0.75 4 0.97 0.9 0.75 0.5 0.1
    $ ./R2R-1.0.6/src/r2r -disable-usage-warning <fileName_cons.sto> <filename_cons.pdf>
  6. Identify conserved nucleotides that can be exploited to design disruptive mutations.
- **Biochemical validation of aptamer candidates**
  1. Perform an elution profile as described in Section C using a single aptamer candidate instead of an RNA pool. For a valid aptamer candidate, we typically expect to observe an increase in signal for only one of the compounds.
  2. There are various biochemical methods that can be used to further validate aptamer-ligand binding, including in-line probing (Soukup and Breaker, 1999), selective 2′-hydroxyl acylation analyzed by primer extension (SHAPE) (Merino et al., 2005), isothermal titration calorimetry (Slavkovic and Johnson, 2023), and surface plasmon resonance (Arney and Weeks, 2022). Our lab prefers in-line probing, for which 5′ ^32^P-labeled RNAs already prepared for the elution profile can be utilized. Refer to previously reported protocols for in-line probing (Regulski and Breaker, 2008). Techniques such as in-line probing and SHAPE that provide structural information are especially useful because they can confirm whether the engineered aptamers retain the structural features of the riboswitch aptamer from which they were derived. With the user’s preferred method for biochemical validation, test candidate aptamers for binding with the target ligand and confirm that disruptive mutant(s) display reduced binding.
- **Grafting aptamer candidates onto their expression platforms and functional testing in cells**
  1. Starting from the sequence of the natural riboswitch that was chosen in part A, remove the entire aptamer domain as well as 2–3 base pairs in the P1 stem located adjacent to the aptamer domain (Figure 1).
  2. Remove the primer-binding regions from the sequence of a biochemically validated aptamer, as well as the sequence of the P1 stem, except for the 2–3 base pairs immediately adjacent to the aptameric core. The number of base pairs retained from the aptamer sequence should be equal to the number that is removed from the expression platform sequence.
  3. Graft the engineered aptamer sequence onto the natural expression platform.
  4. Place a common promoter sequence (e.g., *thiC* for *E. coli, lysC* for *B. subtilis*) upstream of this engineered riboswitch.
  5. Using molecular cloning techniques, install this sequence upstream of a *lacZ* reporter gene within an appropriate plasmid. Another reporter gene, such as green fluorescent protein or luciferase can optionally be used.
  6. Transform this plasmid into a model organism that naturally contains the original riboswitch.
  7. Test the function of the engineered riboswitch by culturing the transformed cells in media supplemented with X-gal (100 μg/mL). If the target ligand does not occur naturally in the cell (e.g., a drug compound), supplement different cultures with and without ligand. Differential blue color between cultures with or without the target ligand indicates that the riboswitch is functional.
  8. Confirm that the directionality of the chosen riboswitch (ON or OFF switch) is reflected in the observed result. Additionally, confirm that the switching effect diminishes between cultures that contain a disruptive mutant construct.

## Data analysis

Analysis of next-generation sequencing data as described in this protocol requires some familiarity with bash commands. Other software, such as FASTAptameR 2.0 (Kramer et al., 2022), can be accessed via the web to facilitate this analysis.

## Validation of protocol

To validate the function of engineered quinine and caffeine riboswitches, we quantitated specific β-galactosidase activity using a Miller assay in the presence and absence of the ligand (Mohsen et al., 2023). Three technical replicates were performed. Statistical analysis was performed with a *t*-test (two-tailed distribution, two sample equal variance).
